# Adenoid Cystic Carcinoma of the Vulva: A Case Report

**DOI:** 10.7759/cureus.56048

**Published:** 2024-03-12

**Authors:** Soumiya Samba, Mohammed Amine Guerrouaz, Ahmed Bensghier, Soufiane Berhili, Mohamed Moukhlissi, Loubna Mezouar

**Affiliations:** 1 Department of Radiation Oncology, Centre Hospitalier Universitaire Mohammed VI, Oujda, MAR; 2 Department of Radiotherapy, Centre Hospitalier Universitaire Mohammed VI, Oujda, MAR; 3 Department of Radiation Oncology, Faculty of Medicine and Pharmacy, Mohammed First University, Oujda, MAR

**Keywords:** vulvar cancer, vulvar carcinoma, bartholin’s gland, vulvar neoplasms, adenoid cystic carcinoma

## Abstract

Adenoid cystic carcinoma (ACC) of the vulva represents a highly uncommon type of female malignancy. Due to the absence of specific treatment guidelines, such cases are typically managed by the treatment protocols for vulvar cancer. Here, we report the case of a 52-year-old woman who presented with a painful right vulvar mass, leading to a diagnosis of ACC of the vulva after biopsy and immunohistochemical analysis. She underwent vulvectomy, bilateral inguinal lymphadenectomy, and targeted radiotherapy, and no evidence of recurrence has been found for three years, with ongoing monitoring for post-radiation effects. This case adds valuable insights into the management of ACC of the vulva and underscores the need for further research and guideline development to optimize care for future patients.

## Introduction

Adenoid cystic carcinoma (ACC) of the vulva is an exceedingly rare entity among female cancers, accounting for less than 0.1% of all female gynecological cancers and 2-7% of vulvar carcinomas [1]. To date, there are only a few cases documented in the literature.

In the head and neck region, these tumors are marked by slow yet aggressive growth, significant perineural spread, and a high risk of local recurrence and distant metastases, leading to a challenging prognosis [1]. Billroth first described ACC as a rare histological subtype of adenocarcinoma, primarily affecting glandular mucosas [2,3]. It notably targets the salivary glands, breasts, and female genital tract, especially the cervix.

ACC in the Bartholin's gland is rare and often misdiagnosed due to its abscess-like symptoms [4-7]. Consequently, the recommended treatment strategy involves comprehensive local surgical removal supplemented by necessary adjuvant radiotherapy [1]. Varela's report supports radiotherapy or chemoradiotherapy as viable for treating Bartholin gland cancers, offering a high survival rate and minimal morbidity [6]. In this report, we present a clinical case admitted to our institution.

## Case presentation

A 52-year-old female patient, one year before admission, presented with a painful right vulvar mass without any other associated signs, evolving in the context of preserved general health status (at that time, she refused to seek a medical consultation). She was admitted to the Department of Gynecology-Obstetrics. Clinical examination revealed a firm, growth located on the right side of the vulva that was sensitive to touch and adhered to adjacent tissues. It measured 2 cm across. No exertion towards the vagina nor to the rectum was detected.

**Figure 1:**
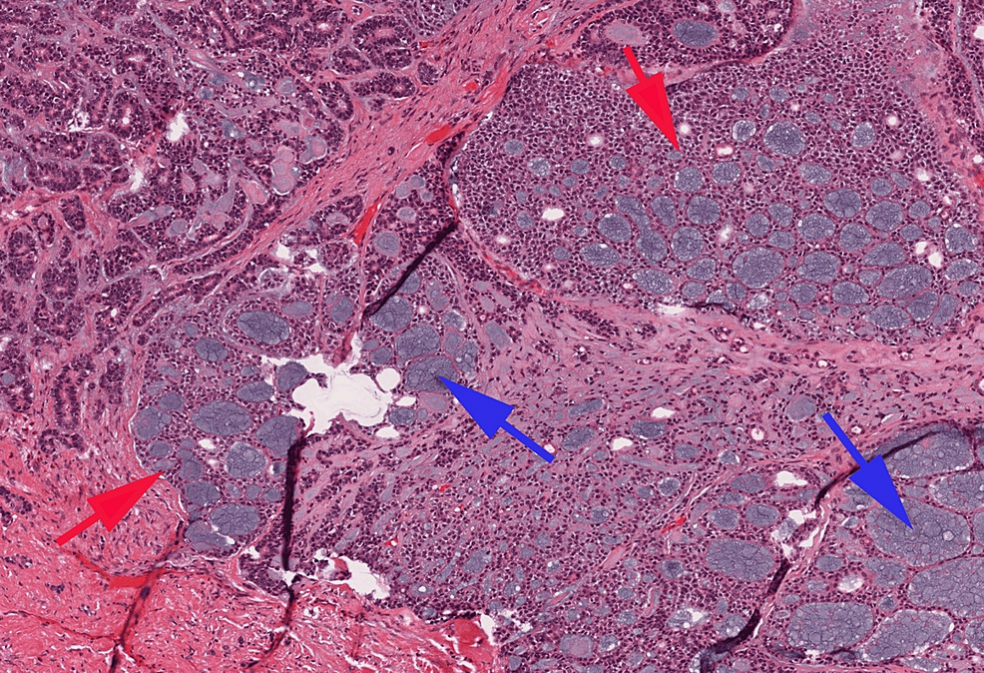
Microphotography showing a cribriform pattern (Red arrows) with the presence of myxoid (Blue arrows) or hyalinized globules (H&E, 100X)

Despite the absence of clear signs of rectal invasion during the recto-vaginal examination, the growth seemed to be attached to the rectum near the entrance. The patient underwent a biopsy of the vulvar lesion. The histopathological examination of the sample revealed a carcinomatous proliferation made of cribriform structures, composed of predominantly myoepithelial cells with myxoid and hyalinized globules (Figure 1).

Further immunohistochemical analysis showed that tumor cells expressed EMA, SMA, CEA, CD117, and vimentin with a high Ki-67 index, compatible with an adenoid cystic carcinoma of the vulva. The resection margins were found to be at 2mm from the tumor.

**Figure 2:**
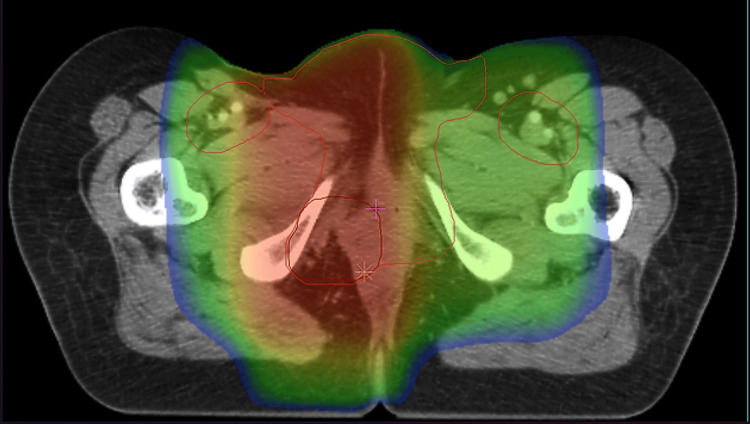
The patient underwent an external three-dimensional conformal radiotherapy at a total dose of 66 Gy in 33 fractions of 2 Gy, five days per week.

Thoracic and abdominopelvic computed tomography (CT) scan was performed and revealed no distant suspicious lesions. On the therapeutic level, the patient underwent a vulvectomy with bilateral inguinal lymphadenectomy, followed by exclusive external three-dimensional conformal radiotherapy at a total dose of 66 Gy in 33 fractions of 2 Gy, five days per week (Figure 2).

**Figure 3:**
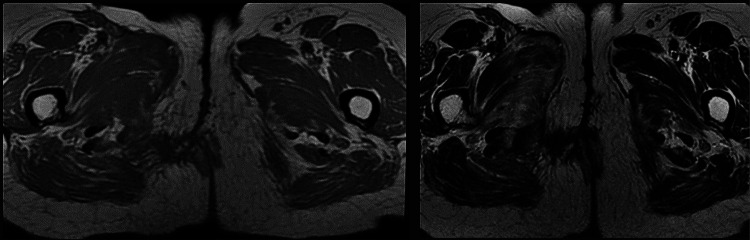
Follow-up MRI performed after completion of radiation therapy showed no local recurrence.

In terms of progression, the radiotherapy was administered without incident. Six months after the completion of irradiation, a follow-up pelvic MRI was conducted (Figure 3), showing no local recurrence according to the RECIST (Response Evaluation Criteria in Solid Tumours) post-radiation therapy response criteria [2].

The patient has been in complete remission for three years and is followed for radiation therapy effects such as post-radiation grade 2 proctitis and mild lymphedema of both lower limbs. The follow-up protocol has been clinical examination every three months and imaging by MRI every six months for two years, then annually.

## Discussion

ACC was first described by Billroth as a histological variant of adenocarcinoma occurring in glandular mucosas [3,4]. It is commonly found in salivary glands, the breast, and the female genital tract; within the latter, it is most frequently located at the cervix. ACC of the Bartholin's gland in the vulva is even rarer. The symptomatology resembles that of a vulvar abscess, manifesting as a painful vulvar mass [5-7], which may be ulcerated, and may or may not be associated with dyspareunia, bleeding, or itching, leading to frequent misdiagnoses as a cyst or abscess [8]. Another significant microscopic feature of this tumor type is the infiltration of perineural spaces. Consequently, many patients experience itching and a burning sensation before the tumor becomes palpable on physical examination [9].

Histologically, ACC is characterized by small cells with scant cytoplasm and dense hyperchromatic nuclei, displaying a basaloid appearance. These cells are arranged in diffuse and massive sheets, cord-like structures, or surrounding cribriform cavities that are either cystic to varying degrees and filled with either slightly eosinophilic hyaline material or basophilic mucin. This may be associated with normal residual tissue of the Bartholin's gland transitioning to cancerous tissue [1,9,10].

Immunohistochemically, this tumor expresses CEA, EMA, CK5/6, CK7, and SMA [7-13]. The immunoreactivity variability documented in the tumor highlights the presence of two distinct components, supported by differential expression of CEA and EMA in the spiradenoma component, in contrast to positivity with CK5/6 and p63 in the cylindroma foci. This immunoreactivity variability across both tumor components contradicts previous studies by Carlesten et al. [8] and Jukic et al. [13], who suggested that spiradenoma and cylindrocarcinoma tumor components share a similar immunohistochemical profile.

The most common site for metastasis is the lung, which is often preceded by a local recurrence. More rarely, bone and brain metastases can occur [7,10,11]. Therapeutic management involves a hemivulvectomy combined with homolateral inguinal lymphadenectomy [10]. In the Korean study by Yoon et al., five patients underwent a hemivulvectomy with homolateral inguinal lymphadenectomy performed in three of them, all of which were negative [11].

Adjuvant treatment includes radiotherapy or chemoradiotherapy, which is particularly beneficial postoperatively. Rosenberg et al. reported five cases of ACC of the vulva, all of which underwent surgical treatment followed by postoperative radiotherapy [14]. There were no recurrences, and three of the patients had postoperative residuals but remained disease-free after 28, 51, and 138 months, indicating that radiotherapy effectively sterilized these tumor residuals.

In the study by Rosenberg et al., external radiotherapy to the the tumor bed and bilateral inguinal areas was delivered at a total dose between 54 Gy and 20 Gy, without specifying the radiotherapy technique nor the used fractionation [14]. In the study by Copland et al., which included 10 patients, there were no local recurrences following postoperative radiotherapy, even in patients with positive margins after surgical resection [10]. Altogether, Rosenberg et al. [14] and Copeland et al. [10] reported 15 cases of postoperative radiotherapy in patients with positive margins, and none developed local recurrence, highlighting the benefit of adjuvant radiotherapy. In another study involving two patients who underwent hemivulvectomy with homolateral inguinofemoral lymphadenectomy, one received postoperative radiotherapy due to positive margins [7]. Neither patient developed local recurrence, though they did experience metastatic relapse. The radiation therapy technique was external beam radiation with a total of 7000 Gy over 35 fractions, delivered to the pelvis, right vulva, and groin area.

Radiotherapy could become the standard treatment for vulvar cancers. In Boston, Lopez-Varela et al. reported 10 cases of Bartholin gland cancer, two of which were ACCs treated with exclusive radiotherapy or chemoradiotherapy [15]. The three-year overall survival rate was 71%, and four patients developed local recurrence after up to 31 months. However, none of the two patients with ACC developed local recurrence, suggesting that radiotherapy could be an effective alternative to surgery for treating Bartholin gland cancers while preserving genital function with very low morbidity. In the study by Lopez-Varela et al., all patients underwent initial radiation treatment, which included external beam radiation therapy targeting the pelvis and a half-boost to established disease sites, with interstitial brachytherapy following in five instances. The external beam radiation was applied using a linear accelerator that emitted high-energy photons. When the groin area was subjected to boost radiation, electron beams were utilized. The interstitial brachytherapy was administered using afterloading methods with Iridium 192 strands. The total radiation doses varied between 45 and 75 Gy [15].

## Conclusions

ACC of the vulva, though rare, presents significant challenges due to its unusual occurrence and the lack of tailored treatment guidelines. This case of a 52-year-old female underscores the potential for successful outcomes through the adaptation of existing vulvar cancer protocols, incorporating surgery and radiotherapy. The patient's journey from diagnosis to treatment and into three years of remission highlights the importance of vigilant clinical assessment, personalized therapeutic approaches, and continuous follow-up to manage this rare malignancy effectively.
